# Supplementary material for: A regulatory circuit of two lncRNAs and a master regulator directs cell fate in yeast
Source: Nat Commun. 2018 Feb 22;9:780. doi: 10.1038/s41467-018-03213-z (PMC5823921; doi:10.1038/s41467-018-03213-z)
Supplement: Supplementary file 1 — Supplementary Information [file 41467_2018_3213_MOESM1_ESM.pdf]

Supplementary Note 1

Mathematical modelling

To understand the dynamical properties of the Ime1 regulation by *IRT1* and *IRT2*, we formulated a mathematical model describing the interactions between *IRT1* and *IRT2* transcription and Ime1 synthesis. The model variables are defined (see list of variables below). The parameters (see list of parameters below) were chosen to reproduce the qualitative properties of the system for the wild-type *IME1* promoter.

List of variables (scaled between 0 and 1)

s	Starvation signal
$I1$	*IRT1* transcription rate
$I2$	*IRT2* transcription rate
I_t	Ime1 transcription rate
I_m	Ime1 mRNA accumulation
I_p	Ime1 protein accumulation

List of parameters used for simulation of the wild-type *IME1* promoter

k_1	5	k_4	1
k_2	0.1	k_5	0.5 /hour
k_3	5	k_6	0.5 /hour

$$s(t) = 1, \qquad \text{for } t > 0 \tag{1}$$

$$I1 = \begin{cases} \dfrac{s}{s + k_1 I2}, & s > 0 \\ 0, & s = 0 \end{cases} \tag{2}$$

$$I2 = \frac{I_p}{I_p + k_2} \tag{3}$$

$$\frac{dI_m}{dt} = \begin{cases} \dfrac{s}{s + k_3 I1} - k_4 I_m, & s > 0 \\ -k_4 I_m, & s = 0 \end{cases} \tag{4}$$

$$\frac{dI_p}{dt} = k_5 I_m - k_6 I_p \tag{5}$$

This model is simulated with the following initial conditions:

$I_{\mathrm{m}}(0) = 0; I_{\mathrm{p}}(0) = 0.$

The first term in Equation (4), $\frac{s}{s+\mathrm{k}_3 I1}$, describes the Ime1 transcription rate (I_{t}).

The network with neither *IRT1* nor *IRT2* corresponds to Equations (1), (4) and (5) and $\mathrm{k}_3 = 0$.

The network without *IRT2* corresponds to Equations (1), (2), (4) and (5) and $k_1 = 0$.

The starvation signal s was modeled as a step input as given in Equation (1). Equation (2) models the *IRT1* transcription rate, $I1$. When the starvation signal s is 0, $I1$ is 0. In Figure 7b and Supplementary Figure 10a starvation signal s was set at 1 at the 0 hour time point for the whole simulation. In Figure 7c and Supplementary Figure 10b we simulated different periods of starvation by setting the starvation signal s at 1 for either 1 hour or 5 hours before setting it back to 0. For positive s, $I1$ is induced by s, but inhibited by the *IRT2* transcription, $I2$. As given in Equation (3), $I2$ is stimulated by Ime1 protein, I_{p}. Equation (4) describes the accumulation of Ime1 mRNA. The first term, $\frac{\mathrm{s}}{\mathrm{s}+\mathrm{k}_3\mathrm{I1}}$, describes the Ime1 transcription rate (I_{t}) and the second term, $-\mathrm{k}_4\mathrm{I}_{\mathrm{m}}$, describes the Ime1 mRNA degradation. Equation (5) models Ime1 protein, where the first term, $\mathrm{k}_5 I_{\mathrm{m}}$, describes Ime1 protein production from Ime1 mRNA and the second term, $-\mathrm{k}_6 I_{\mathrm{p}}$, describes the Ime1 protein degradation.

The mathematical model was simulated in MATLAB 2017a using ode15s and ode45 functions. The code is available upon request.

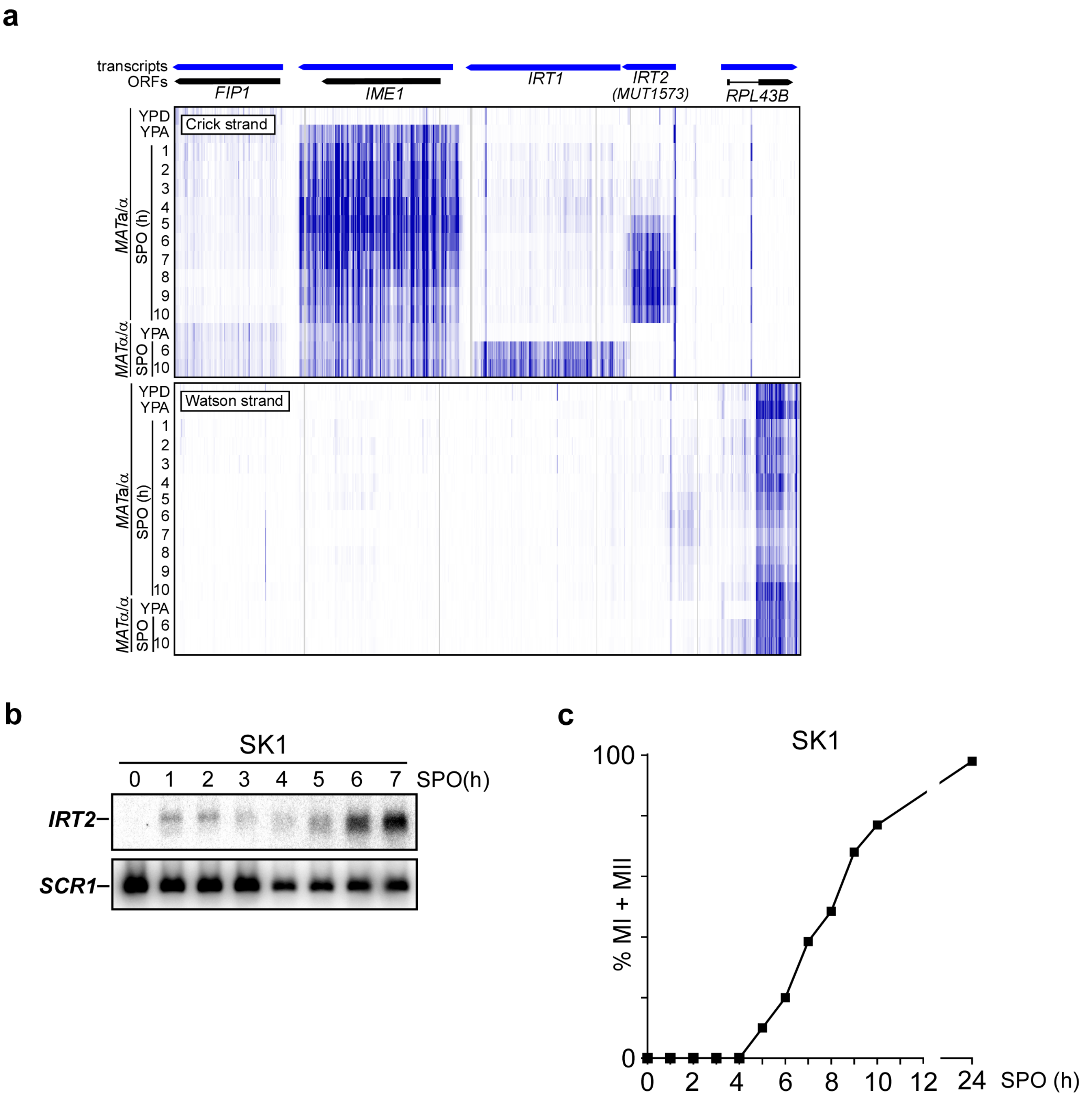

Supplementary Figure 1. The lncRNA *IRT2* is expressed during entry into meiosis
(a) Data from Lardenois *et al.* plotted in the integrative genomics viewer[1,2]. SK1 *MAT*a/α and *MAT*α/α diploid cells were grown in rich medium (YPD), pre-sporulation medium (YPA), and transferred to sporulation (SPO) (see Lardenois *et al.* for details)[1]. The expression profiles of the *IME1* locus, including the lncRNAs *IRT1* and *IRT2*, and adjacent genes from the Watson and Crick strands are displayed. The indicated time points in SPO, YPD, and YPA are shown. Blue indicates expression and white indicates no expression. **(b)** *IRT2* expression in SK1 diploid cells during entry into meiosis (FW1511). Cells were grown till saturation in rich medium, shifted and grown in pre-sporulation medium (BYTA), and transferred to SPO. Samples were taken at the indicated time points. A probe directed to upstream region in the *IME1* promoter was used to detect *IRT2* by northern blot. The blot was also probed for *SCR1*, which was used as a loading control. **(c)** Kinetics of meiotic divisions (MI+MII) in wild-type (FW1511). Cells were grown as described in (b), fixed, stained, and DAPI masses of n=200 cells were counted. Cells with two or more masses were considered to have completed at least one meiotic division.

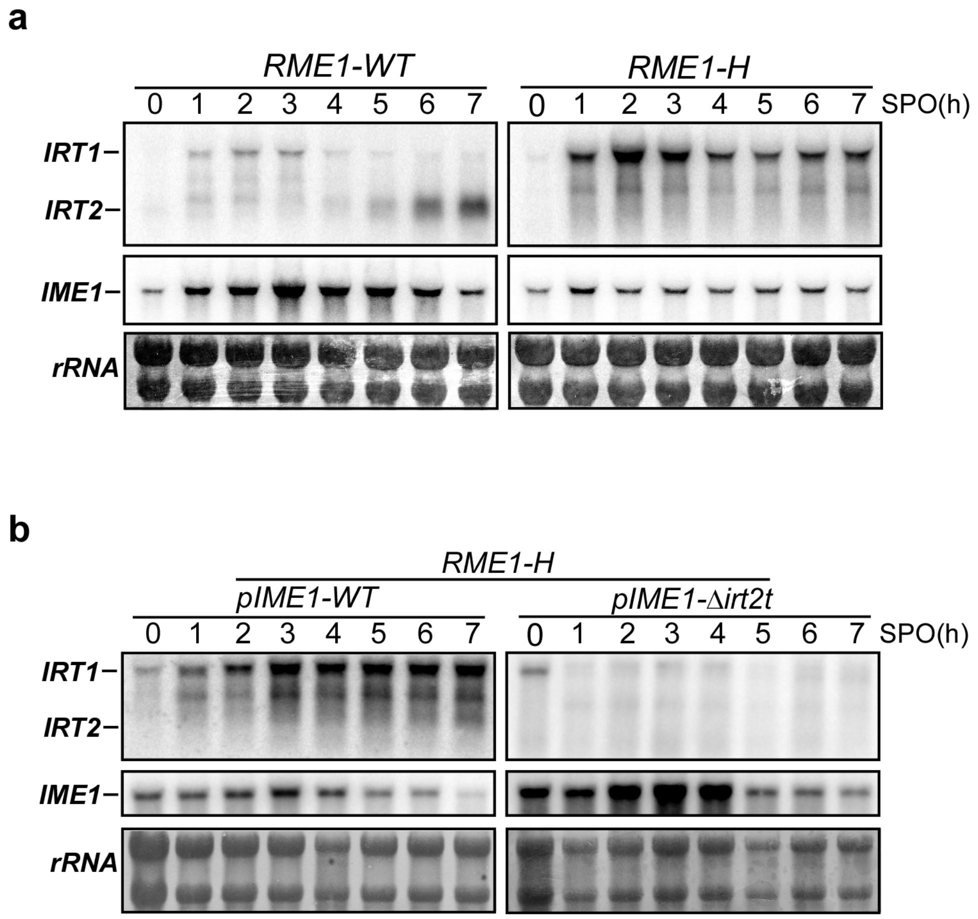

Supplementary Figure 2. Transcription of the lncRNA *IRT2* promotes meiosis
(a) Expression of *IRT1*, *IRT2* and *IME1* during entry in meiosis detected by northern blot. SK1 wild-type (*RME1-WT*, FW1511) and *RME1-H* (FW1196) diploid cells were grown till saturation in YPD, diluted to pre-sporulation medium (BYTA), and subsequently shifted to SPO medium. Samples were taken at the indicated time points. Radionuclide labelled DNA probes were used to detect *IRT1* and *IRT2*, or *IME1*. As loading control ribosome RNA (rRNA) is shown. **(b)** Similar as (a) except that cells wild-type for *IME1* (*pIME1-WT*) and cells lacking a large part of the *IRT2* sequence (*pIME1-Δirt2t*) (FW1327) in the background of *RME1-H* were used for the analysis.

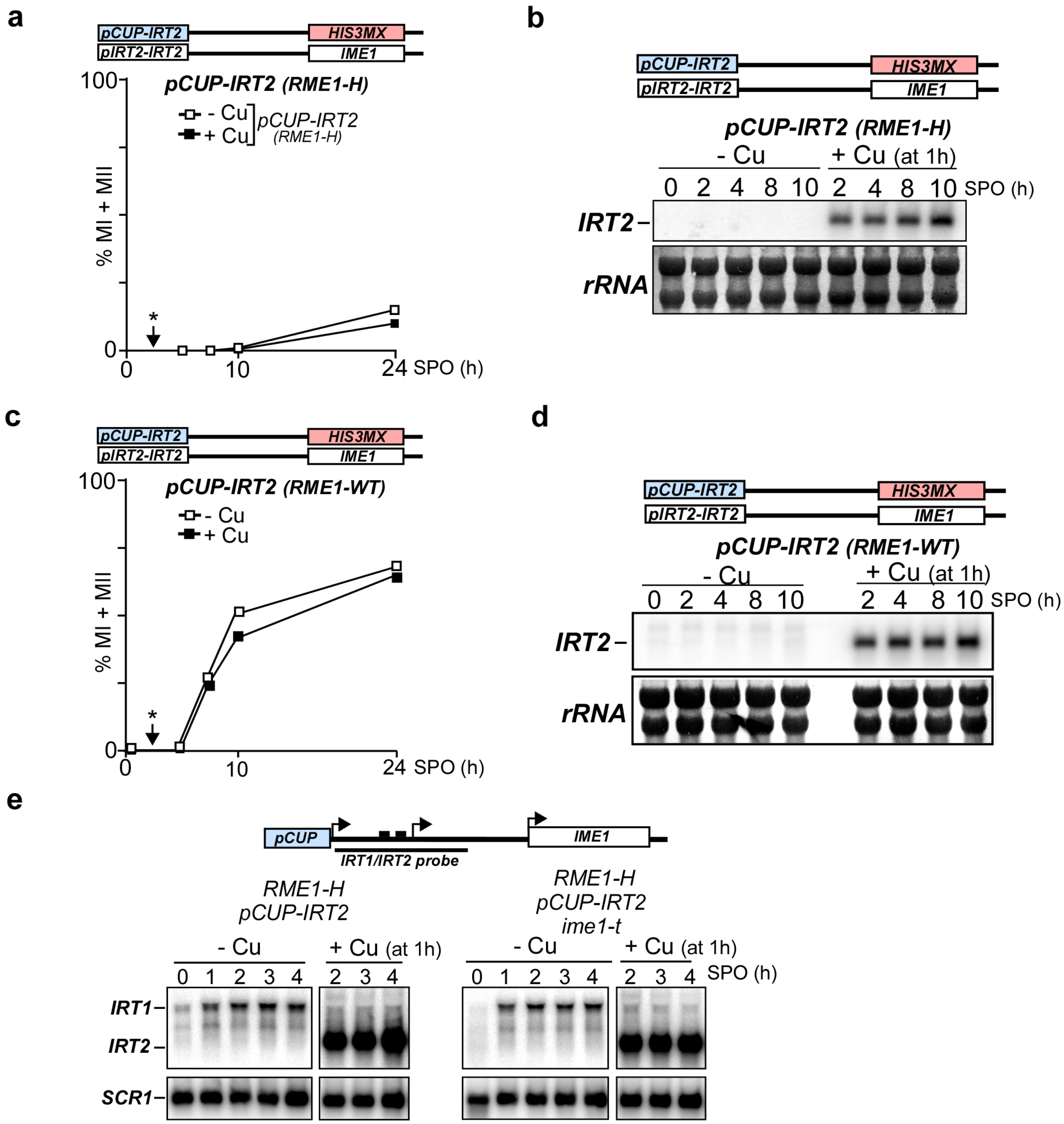

Supplementary Figure 3. *IRT2* promotes entry into meiosis *in cis* of *IME1*

(a) Quantification of cells that completed meiotic divisions. A diploid SK1 strain harboring *RME1-H* together with a heterozygous *IME1* locus of which one copy harboring *pCUP1-IRT2* directly upstream of *ime1*Δ (FW2755) and the other copy *IME1* wild-type was used for the analysis. Cells were grown till saturation in rich medium (YPD), diluted and grown overnight in pre-sporulation medium (BYTA), shifted to sporulation medium (SPO) and were either untreated (-Cu) or treated with copper sulfate (+Cu, 25 μM) after one hour in SPO. Samples were taken at the indicated time points, fixed, stained, and DAPI masses of n=200 cells were counted. * treatment with copper sulfate. **(b)** Similar as (a) except that *IRT2* expression was detected by northern blot. **(c)** Similar as (a) except that the wild-type *RME1* allele (*RME1-WT*) was used (FW2709). **(d)** Similar analysis as (b), but strains described in (c) were used. **(e)** *IME1* is not required for *IRT1* mediated repression by *IRT2*. *RME1-H* diploid cells with *IRT2* expressed from the *CUP1* promoter (*pCUP-IRT2*) combined with a 3' end mutation in *IME1* (*ime1-t*) (FW5924), or not (FW2385) were grown till saturation in YPD. Subsequently, cells were diluted to BYTA, shifted to SPO, and were either untreated (-Cu) or treated with copper sulfate (+Cu, 25 μM) after one hour in SPO. Samples were taken at the indicated time points. *IRT2* and *IRT1* expression were detected by northern blot. As a loading control *SCR1* is shown.

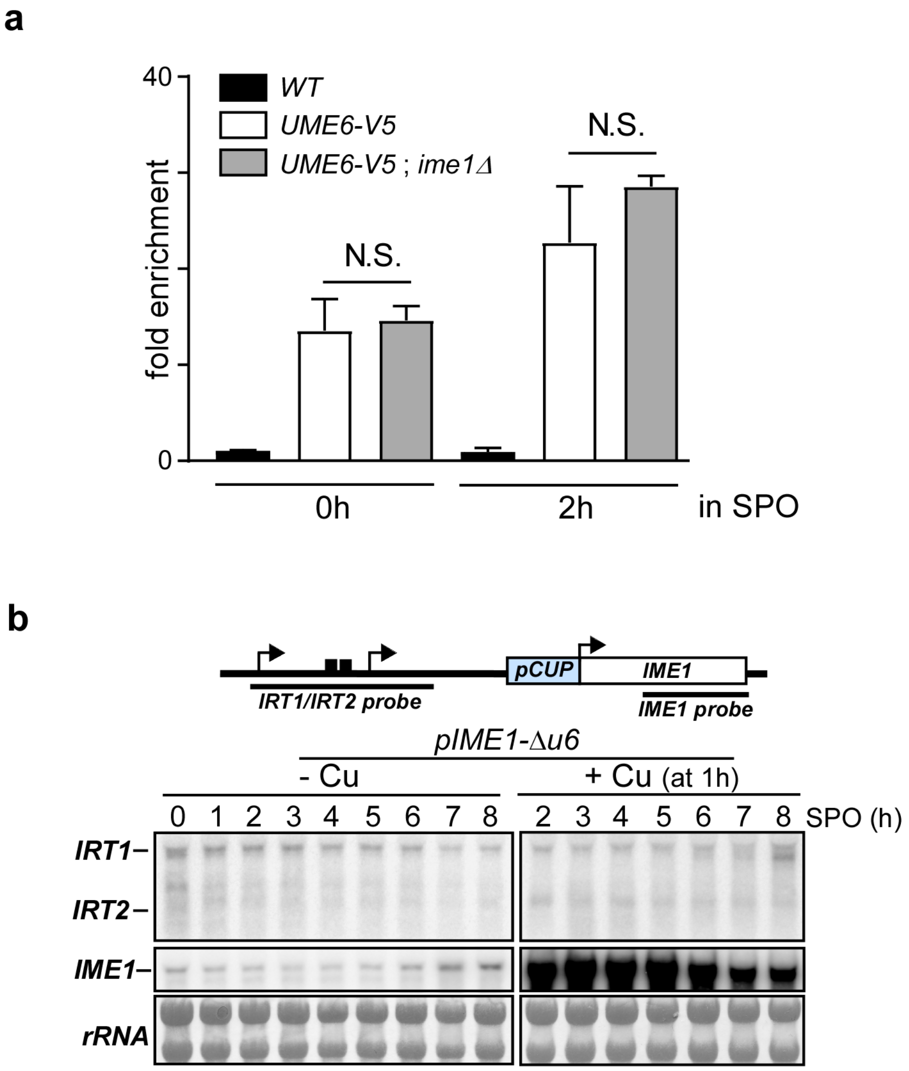

Supplementary Figure 4. Ume6 and Ime1 control *IRT2* expression
(a) Binding of Ume6 to the *IME1* promoter is not dependent on *IME1.* Diploid cells that were either wild type (FW1511) or harboring a V5 tagged Ume6 (Ume6-V5) (FW1208), or Ume6-V5 with an *IME1* deletion (*ime1*Δ, FW5918) were grown to enter meiosis. Chromatin immunoprecipitation was performed using anti-V5 antibodies. Signals were normalized over the silent mating-type locus. Means ±SEM of n=3 experiments are shown. There was no significant difference (N.S., $p > 0.05$, Student's *t*-test) between Ume6-V5 and Ume6-V5, *ime1*Δ **(b)** *IRT2*, *IRT1* and *IME1* expression during entry into meiosis detected by northern blot. Diploid cells with *IME1* expressed from the *CUP1* promoter combined with a deletion in the Ume6 binding site (*pIME1-Δu6*) (FW2842) were grown till saturation in YPD, diluted BYTA, shifted to SPO, and were either untreated (-Cu) or treated with copper sulfate (+Cu, 25 μM) after one hour in SPO. Samples were taken at the indicated time points. As loading control ribosome RNA (rRNA) is shown.

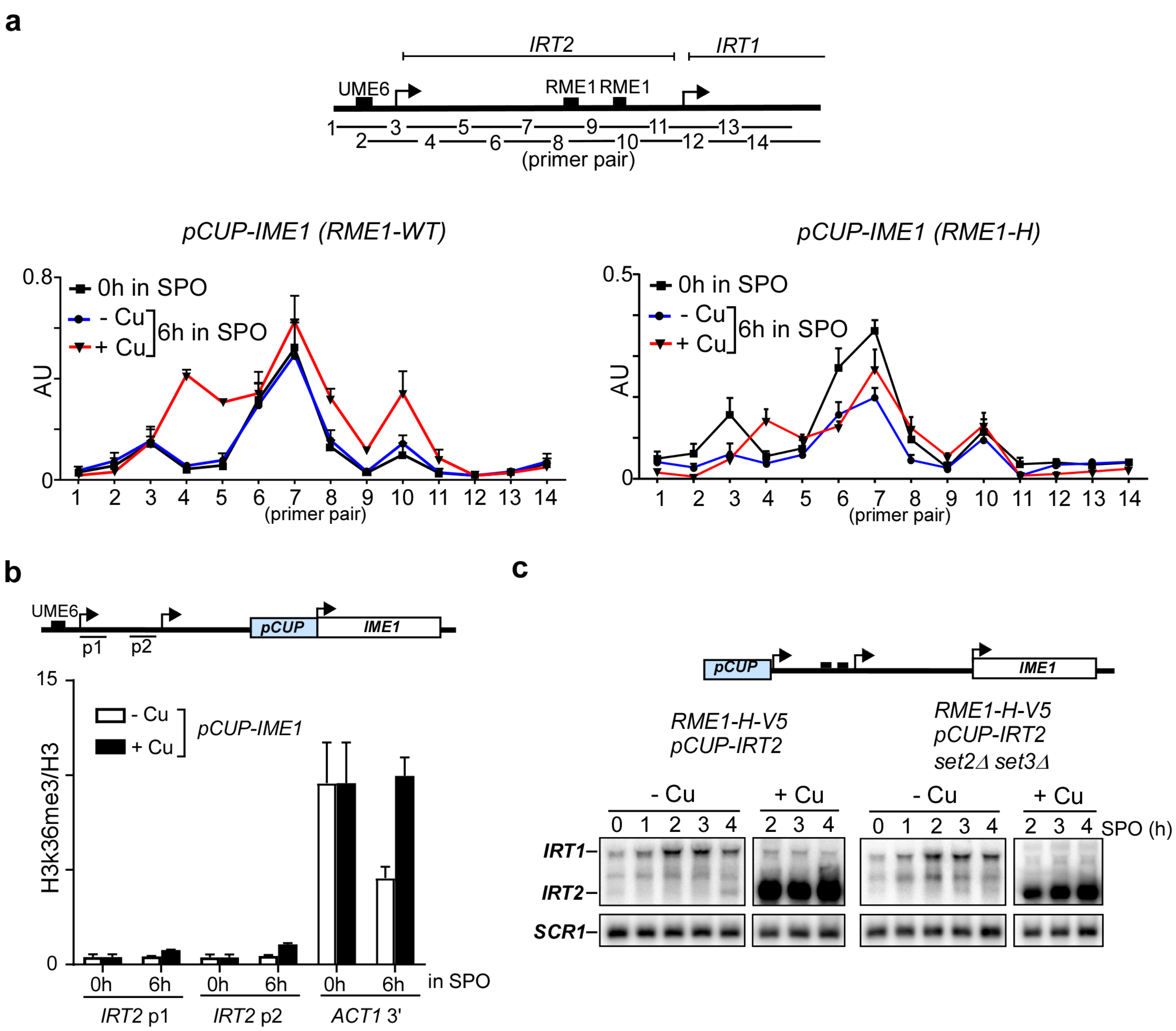

Supplementary Figure 5. Ime1 changes the chromatin state upstream in the *IME1* promoter via *IRT2*
(a) *IME1* mediated activation of *IRT2* alters the chromatin state upstream in the *IME1* promoter. *pCUP-IME1* combined with *RME1-WT* (FW3006) or *pCUP-IME1* combined with *RME1-H* (FW2270) cells were induced to enter meiosis in the absence (-Cu) or presence (+Cu) of *IME1* expression, fixed with formaldehyde, and extracted chromatin was treated with micrococcal nuclease (MNase). Mono-nucleosome DNA fragments were isolated and quantified using 14 primers pairs across *IRT2* region of the *IME1* promoter. The signals were normalized to a no MNase input. The means ±SEM of n=3 experiments is shown. **(b)** Histone H3 lysine 36 trimethylation (H3K36me3) is not enriched at the *IRT2* locus in the absence or presence of *IRT2* transcription. Cells harboring *pCUP-IME1* (FW3006) were grown as described in (a). Samples were formaldehyde crosslinked and chromatin immunoprecipitation was performed using antibodies directed against H3K36me3 and histone H3. Two primer pairs (p1 and p2) were used to detect H3K36me3 enrichment at the *IRT2* locus. As a positive control a primer pair directed against the 3' end of the *ACT1* gene was used. The signals were normalized over histone H3. The mean ±SEM of n=2 experiments is shown. **(c)** *IRT2*, *IRT1* and *SCR1* expression during entry into meiosis detected by northern blot. Diploid cells harboring *RME1-H-V5* allele together with *pCUP-IRT2* that were either wild type for *SET2 SET3* (FW2060) or contained the *set2*Δ *set3*Δ double mutant (FW2241) were grown till saturation in YPD. Subsequently cells were diluted to BYTA, shifted to SPO, and were either not treated (-Cu) or treated with copper sulfate (+Cu, 25 μM) after one hour in SPO. Samples were taken at the indicated time points. As a loading control *SCR1* is shown.

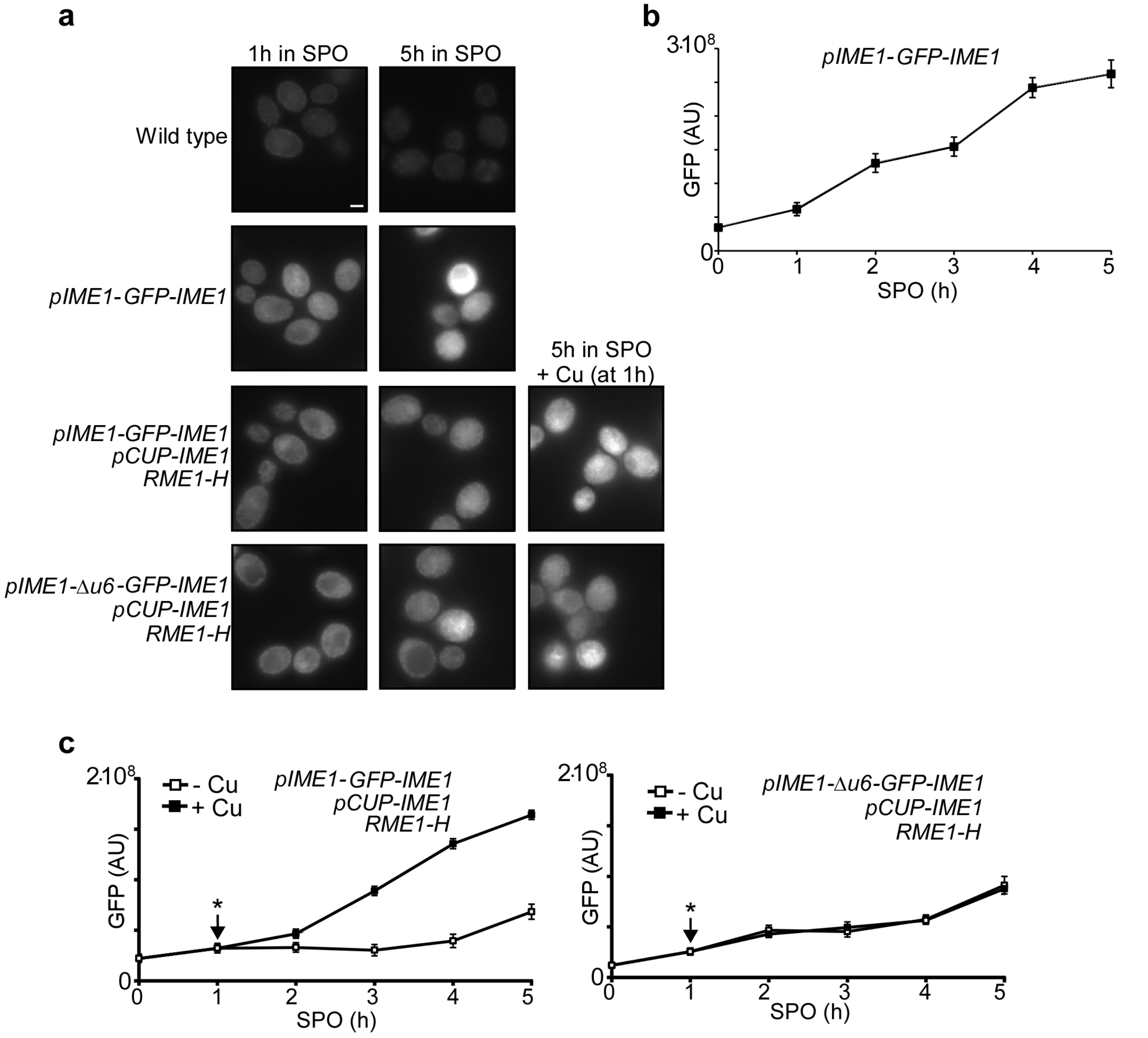

Supplementary Figure 6. *IME1* promotes its own expression via *IRT2*
(a) Representative images. Wild type (1511), *pIME1-GFP-IME1* (FW4653), and cells containing *RME1-H* and one copy of *pCUP-IME1*, one copy of *pIME1-GFP-IME1* (FW5291) or *pIME1-Δu6-GFP-IME1* (FW5295) were induced to enter meiosis. In short, cells were grown in YPD till saturation, diluted and grown in BYTA overnight, shifted to SPO, and were either untreated (-Cu) or treated with copper sulfate (+Cu, 5 μM) after one hour in SPO. The scale bar in right bottom corner represents 2 μm. **(b)** Quantification of *pIME1-GFP-IME1* signals during entry in meiosis. Cells were grown as described in (a), imaged, quantified, and background corrected. We also measured the auto fluorescence signal of wild-type cells for each time point, and subtracted the signal from GFP signal (See Methods). The values displayed in the graph were corrected for background and auto fluorescence. Means ± error bars that represent the 95% confidence interval are shown. **(c)** Similar as (b), except that the strains, FW5291 and FW5295 described in (a), were used for the analyses. * treatment with copper sulfate.

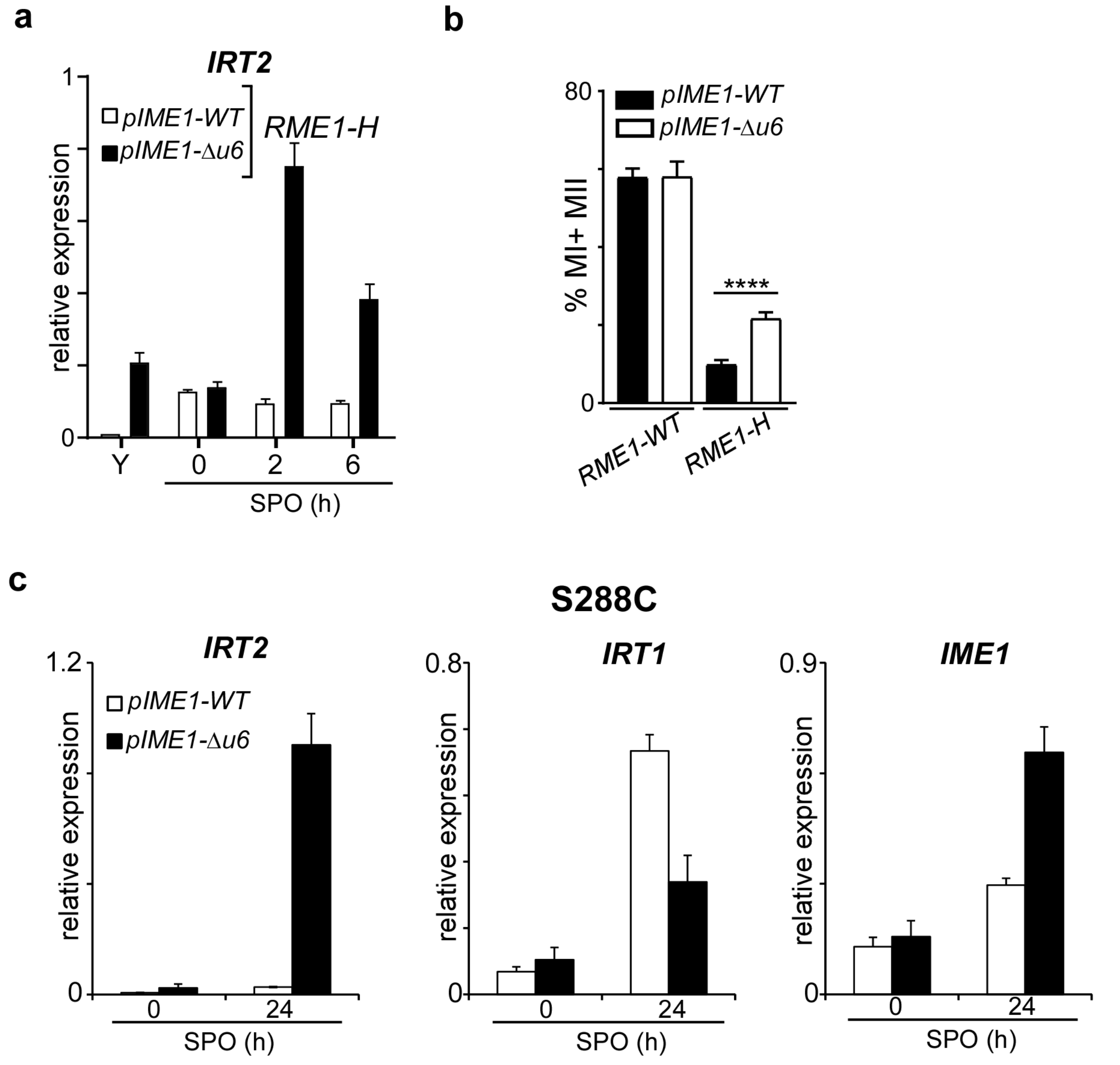

Supplementary Figure. 7. The Ume6 binding site prevents aberrant *IRT2* transcription
(a) Quantification of *IRT2* expression during entry in meiosis. Diploid cells harboring *RME1-H* combined with wild-type *IME1* (*pME1-WT*) (FW1196) or *pIME1-Δu6* (FW2662) were grown till saturation in YPD, and transferred to SPO medium. Samples were taken at the indicated time points. *IRT2* levels were quantified by reverse transcription and quantitative PCR, and the signals were normalized to *ACT1*. Means ±SEM of two biological experiments are shown. **(b)** Quantification of cells that completed meiotic divisions. Wild-type cells (FW1511) and cells harbouring *pIME1-Δu6* (FW2449), *RME1-H* (FW1196) or both alleles (FW2662) were grown as described in (a). Cells were fixed in ethanol after 48h in SPO, and DAPI masses were counted for at least 200 cells. The means ±SEM of at least n=5 experiments are shown. **** $p < 0.0001$ (Student's *t*-test). **(c)** Quantification of *IRT2*, *IRT1* and *IME1* expression during entry in meiosis in S288C. Wild-type (*pIME1-WT*) (FW631) and *pIME1-Δu6* (FW1390) cells were grown as described in (a). Samples were taken at 0h and after 24h in SPO. *IRT1*, *IRT2*, and *IME1* levels were quantified by reverse transcription and quantitative PCR, and the signals were normalized to *ACT1*. The means ±SEM of n=3 experiments are shown.

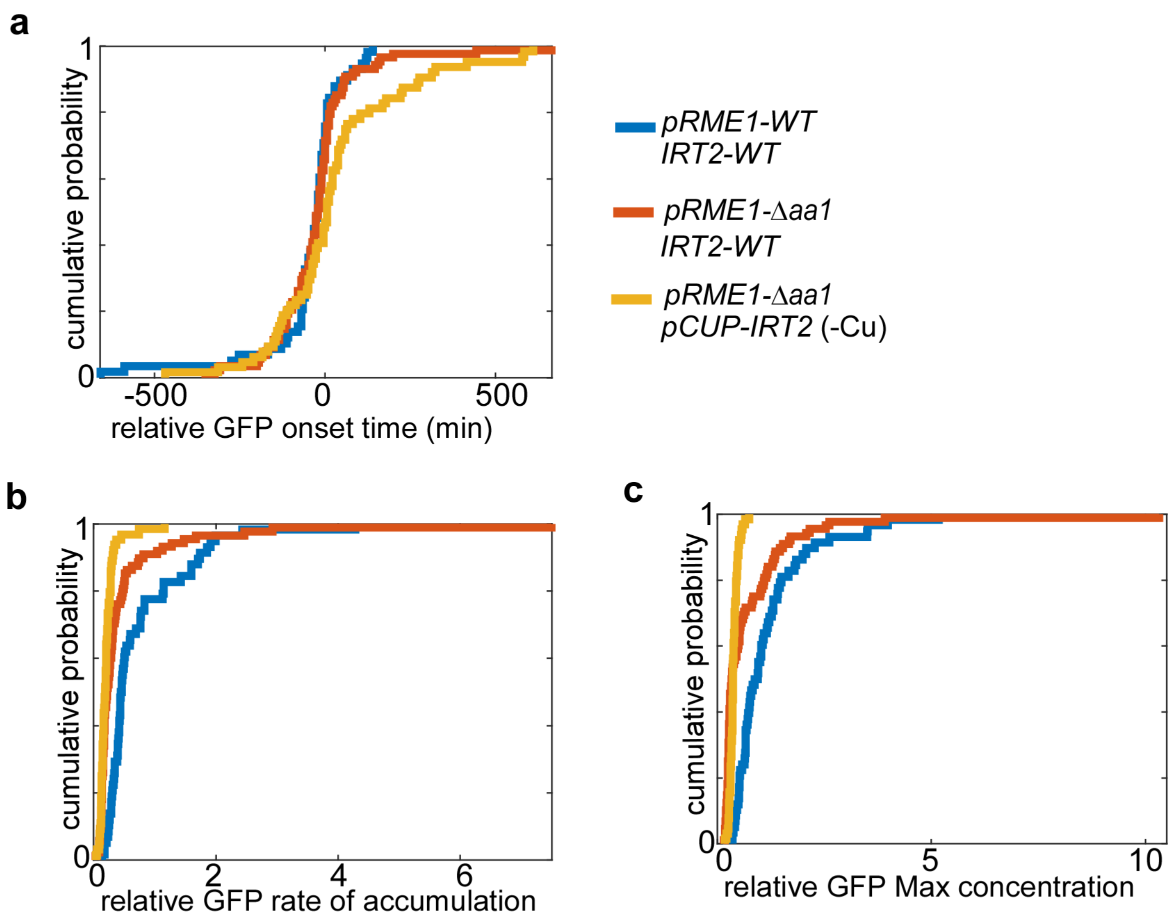

Supplementary Figure 8. Cumulative probability plots from data of time lapse microscopy
Cumulative probability plots from the data obtained with the time lapse microscopy experiments. Cells with one copy of *pIME1-GFP-IME1* and one copy of *pIME1-Δirt2-mCherry*, and either *pRME1-WT* (FW4843), *pRME1-Δaa1* (FW4844) or *pRME1-Δaa1, pCUP-IRT2* (FW5051) were imaged. GFP and mCherry fluorescence signals were quantified. Cumulative probability plots for onset time **(a)**, rate of accumulation **(b)** and maximum intensity **(c)** of GFP relative to mCherry signal are shown (see Methods for details).

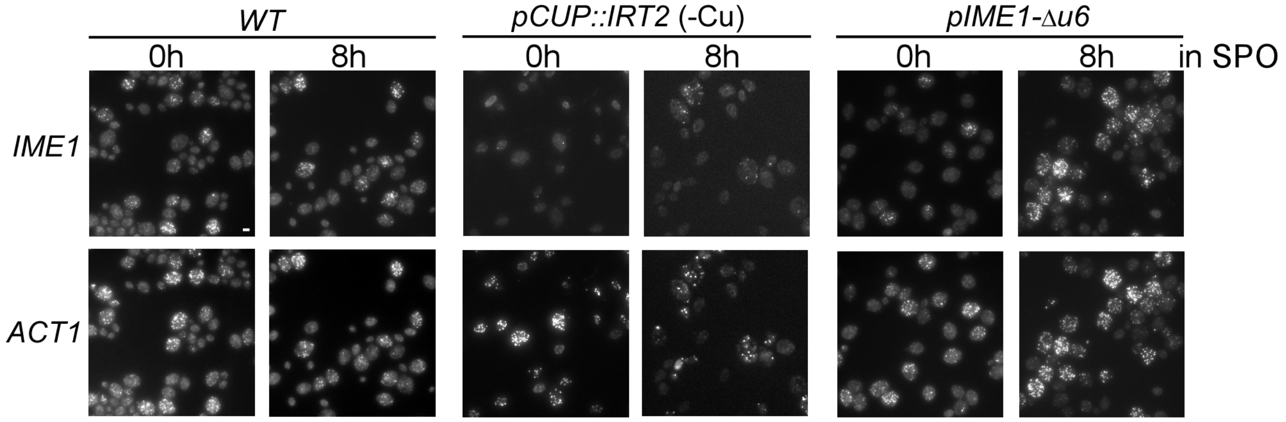

Supplementary Figure 9. *IME1* expression in single cells
Representative images used to quantify *IME1* transcripts in S288C wild-type (WT) (FW631), *pCUP-IRT2* (FW2668) and *pIME1-Δu6* (FW1390) strains using single molecule RNA FISH. Cells were grown in YPD till saturation, shifted to SPO, samples were taken at the indicated time points, formaldehyde fixed, and hybridized with probes directed against *IME1* (AF594) and *ACT1* (Cy5) (see Methods for details). The scale bar in right bottom corner represents 2 μm.

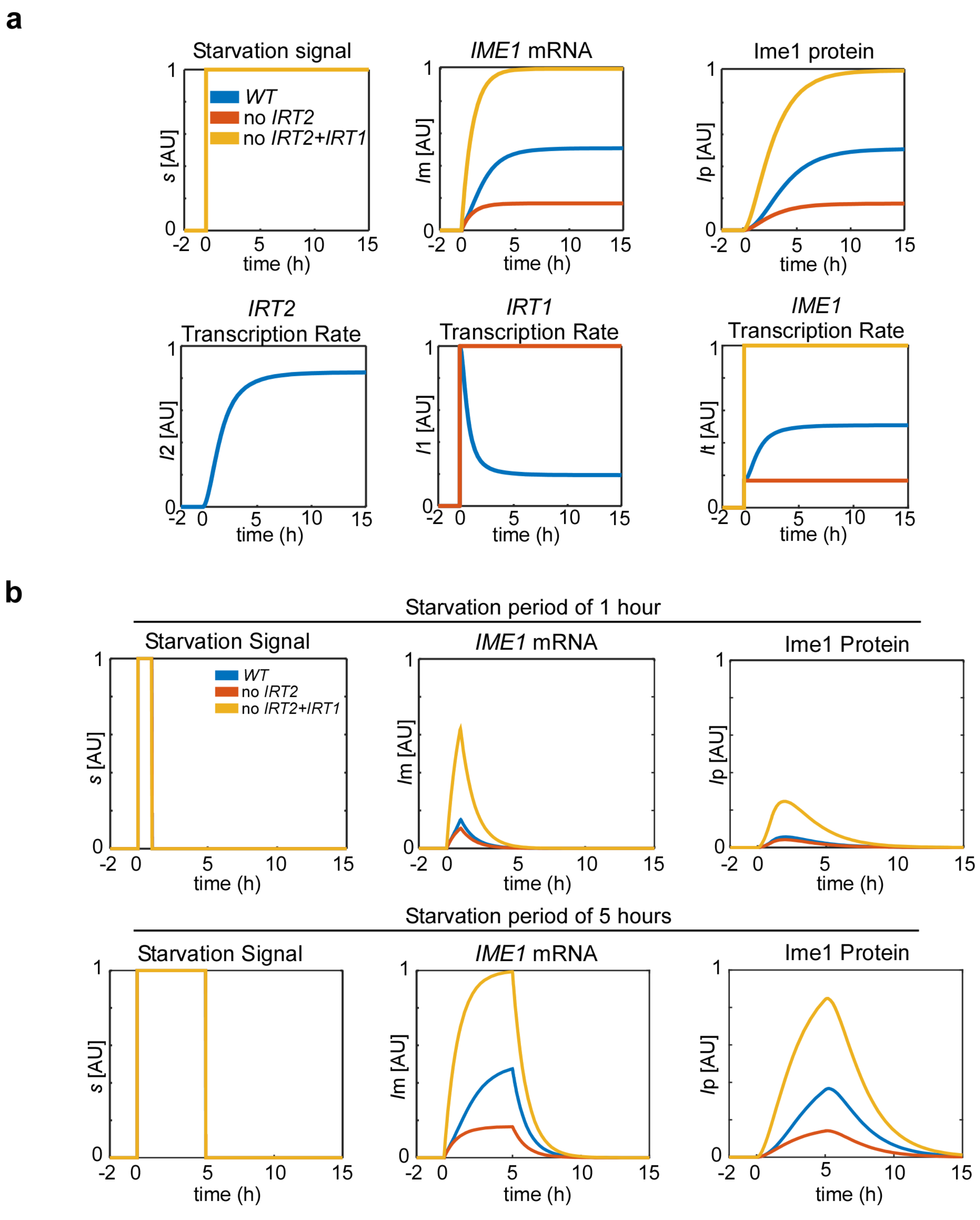

Supplementary Figure 10. Mathematical modeling of *IME1* feedback cascade
(a) Simulation of *IME1* mRNA accumulation (*I*m), Ime1 protein accumulation (*I*p) and *IRT2* (*I*2), *IRT1* (*I*1) and *IME1* (*I*t) transcription rates prior (*s*=0, before 0 hours) and during starvation (*s*=1) for the wild type *IME1* promoter, in the absence of *IRT2* (no *IRT2*), or in the absence of *IRT1* and *IRT2* (no *IRT2*+*IRT1*). The y-axis displays the level of *s*, *I*t, *I*1, *I*2, *I*m or *I*p in arbitrary units (AU) scaled between 0 and 1. **(b)** Simulation of *IME1* mRNA (*I*m) and protein (*I*p) accumulation during starvation periods of 1 hour and 5 hours.

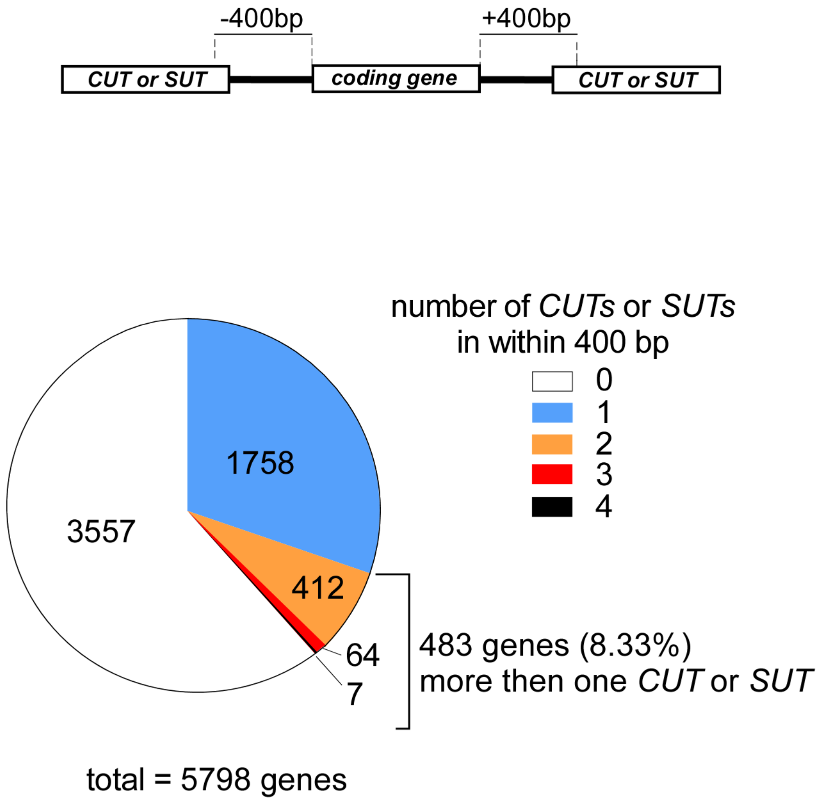

Supplementary Figure 11. Quantification of the number of genes that show local expression of *CUTs* or *SUTs*
We used a dataset from Wery *et al*. to quantify the number of genes that display local expression of cryptic unstable transcripts (*CUTs*) or stable unannotated transcripts (*SUTs*)[3]. We used a cut-off of 400 bp upstream and downstream of the gene sequence (+/- 400 bp). *CUTs* or *SUTs* that overlapped with at least 1 bp of the gene sequence +/- 400 bp were scored positive in the analysis.

Figure 1b

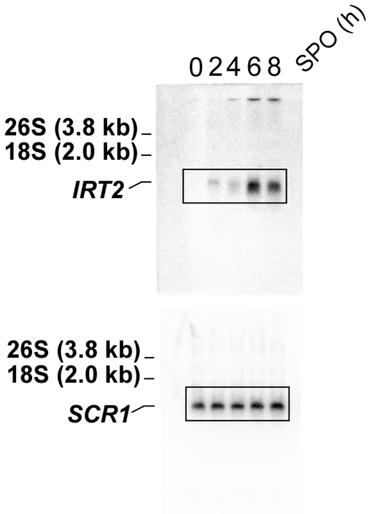

Figure 2a

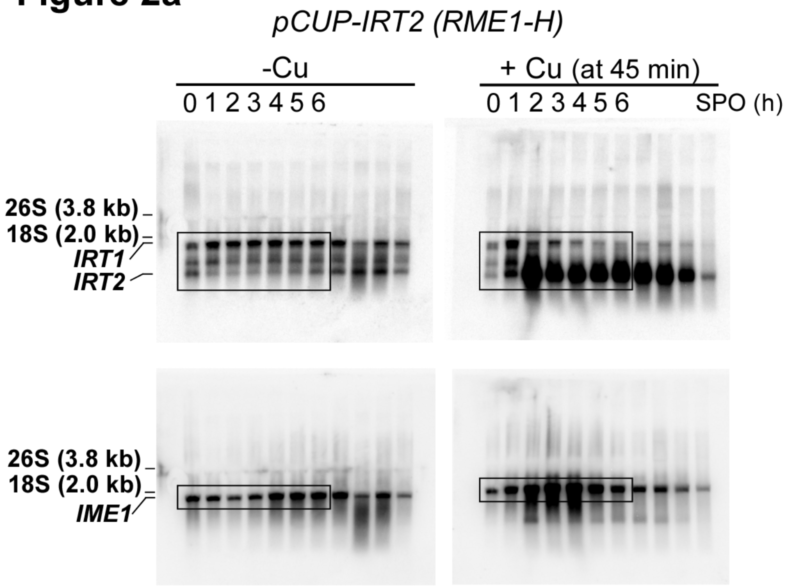

Figure 3c

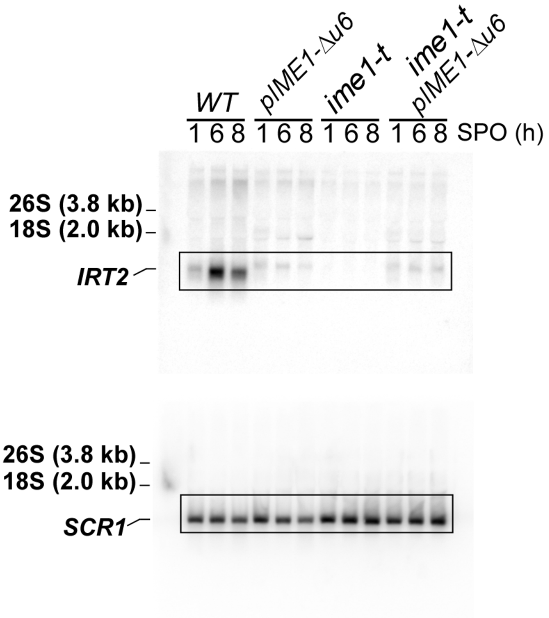

Figure 3d and Supplementary figure 4b

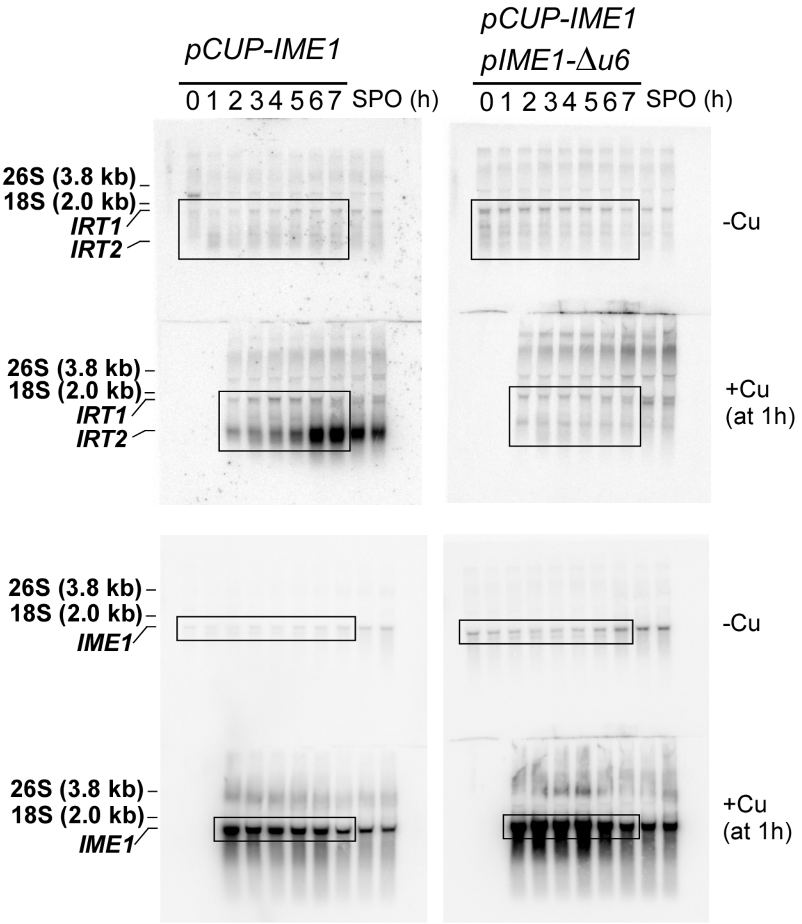

Supplementary Figure 12. Uncropped northern blots 1/2

Figure 4c

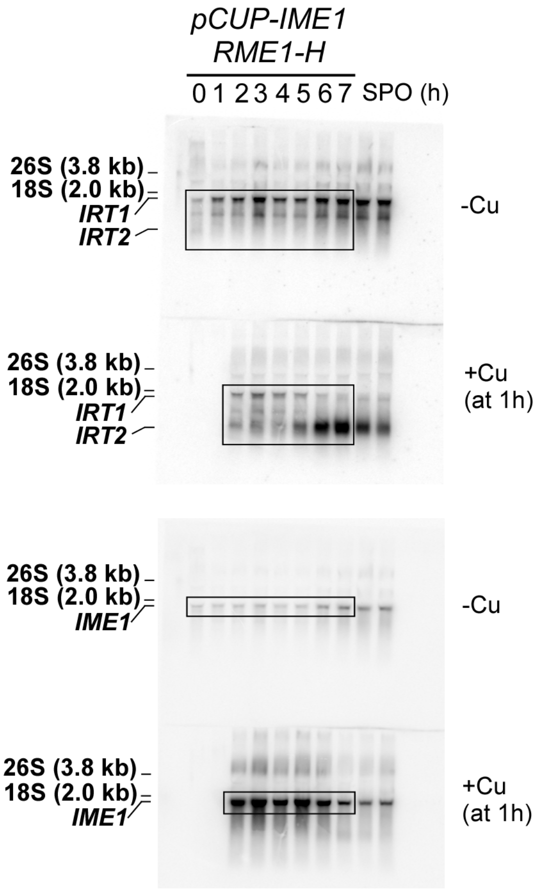

Figure 4d

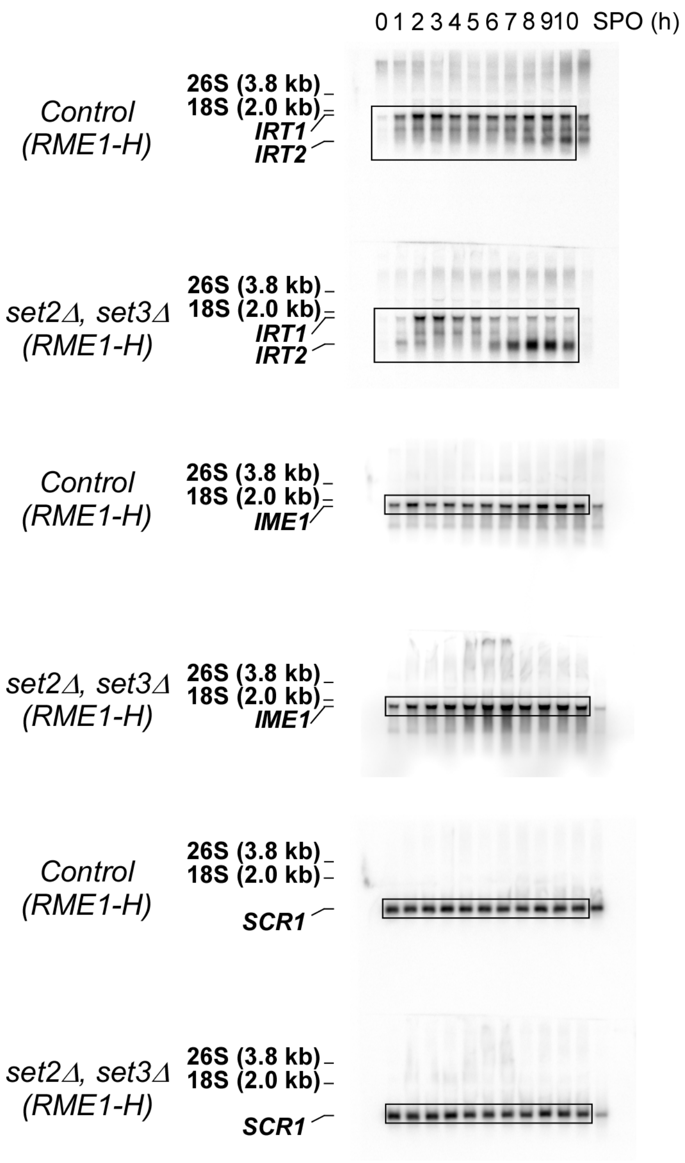

Supplementary Figure 3b

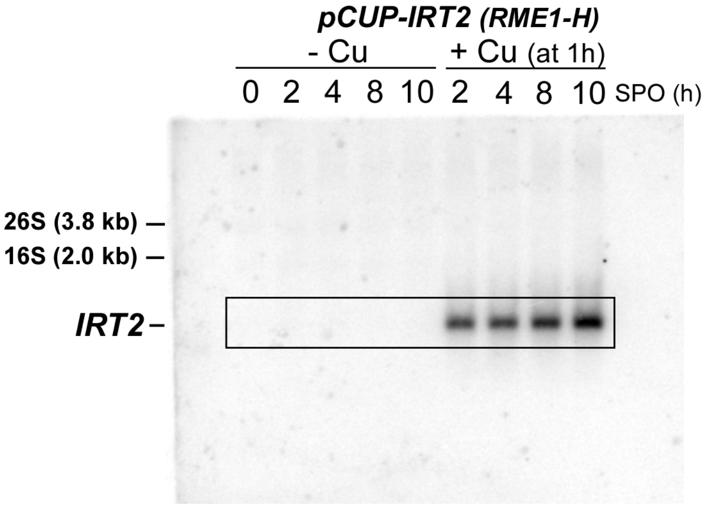

Supplementary Figure 3d

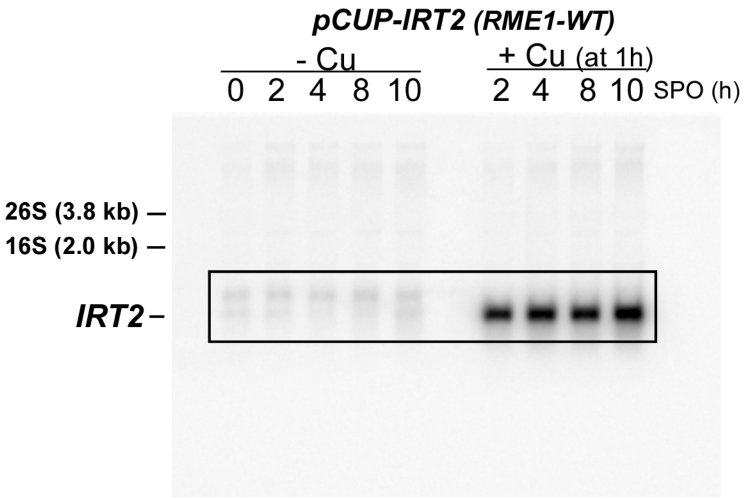

Supplementary Figure 12. Uncropped northern blots 2/2

Supplementary Table 1. Strain genotypes

Strain number	Genotype	background
FW631	*MATa, his3D1, leu2D0, met15D0, ura3D0,*<br>*MATα, his3D1, leu2D0, met15D0, ura3D0,*	*S288C*
FW1497	*MATa, his3D1, leu2D0, met15D0, ura3D0, rme1Δ::HIS3*<br>*MATα, his3D1, leu2D0, met15D0, ura3D0, rme1Δ::HIS3*	*S288C*
FW2668	*MATa, his3D1, leu2D0, met15D0, ura3D0, KanMX-pCUP-IRT2*<br>*MATα, his3D1, leu2D0, met15D0, ura3D0, KanMX-pCUP-IRT2*	*S288C*
FW1390	*MATa, his3D1, leu2D0, met15D0, ura3D0, KanMX::pIME1-Δu6*<br>*MATα, his3D1, leu2D0, met15D0, ura3D0, KanMX::pIME1-Δu6*	*S288C*
FW1511	*MATa, ho::LYS2, lys2, ura3, leu2::hisG, his3::hisG, trp1::hisG*<br>*MATα , ho::LYS2, lys2, ura3, leu2::hisG, his3::hisG, trp1::hisG*	*SK1*
FW1196	*MATa, ho::LYS2, ura3, leu2::hisG, his3::hisG, trp1::hisG, TRP1-RME1-H*<br>*MATα, ho::LYS2, ura3, leu2::hisG, his3::hisG, trp1::hisG, TRP1-RME1-H*	*SK1*
FW2340	*MATα, ho::LYS2, lys2, ura3, leu2::hisG, his3::hisG, trp1::hisG, rme1Δ::HIS3*<br>*MATa, ho::LYS2, lys2, ura3, leu2::hisG, his3::hisG, trp1::hisG, rme1Δ::HIS3*	*SK1*
FW2476	*MATα, ho::LYS2, lys2, ura3, leu2::hisG, his3::hisG, trp1::hisG, KanMX-pCUP-IRT2, rme1Δ::HIS3*<br>*MATa, ho::LYS2, lys2, ura3, leu2::hisG, his3::hisG, trp1::hisG, KanMX-pCUP-IRT2, rme1Δ::HIS3*	*SK1*
FW2385	*MATα, ho::LYS2, lys2, ura3, leu2::hisG, his3::hisG, trp1::hisG, KanMX-pCUP-IRT2, TRP1-RME1-H*<br>*MATa, ho::LYS2, lys2, ura3, leu2::hisG, his3::hisG, trp1::hisG, KanMX-pCUP-IRT2, TRP1-RME1-H*	*SK1*

FW5254	MATα, ho::LYS2, lys2, ura3, leu2::hisG, his3::hisG, trp1::hisG, KanMX-pCUP-IRT2<br>MATa, ho::LYS2, lys2, ura3, leu2::hisG, his3::hisG, trp1::hisG, KanMX-pCUP-IRT2	SK1
FW2060	MATα, ho::LYS2, lys2, ura3, leu2::hisG, his3::hisG, trp1::hisG, KanMX-pCUP-IRT2, TRP1-RME1-H-3V5-HIS3<br>MATa, ho::LYS2, lys2, ura3, leu2::hisG, his3::hisG, trp1::hisG, KanMX-pCUP-IRT2, TRP1-RME1-H-3V5-HIS3	SK1
FW2709	MATα, ho::LYS2, lys2, ura3, leu2::hisG, his3::hisG, trp1::hisG<br>MATa, ho::LYS2, lys2, ura3, leu2::hisG, his3::hisG, trp1::hisG, KanMX-pCUP-IRT2, ime1Δ::HIS3	SK1
FW2755	MATα, ho::LYS2, lys2, ura3, leu2::hisG, his3::hisG, trp1::hisG, KanMX-pCUP-IRT2, ime1Δ::HIS3, TRP1-RME1-H<br>MATa, ho::LYS2, lys2, ura3, leu2::hisG, his3::hisG, trp1::hisG, TRP1-RME1-H	SK1
FW1327	MATα, ho::LYS2, lys2, ura3, leu2::hisG, his3::hisG, trp1::hisG, irt2Δ::HIS3, TRP1-RME1-H<br>MATa, ho::LYS2, lys2, ura3, leu2::hisG, his3::hisG, trp1::hisG, irt2Δ::HIS3, TRP1-RME1-H	SK1
FW2000	MATα, ho::LYS2, ura3, leu2::hisG, his3::hisG, trp1::hisG, UME6-3V5-HIS3, KanMX::pIME1-Δu6	SK1
FW2978	MATα, ho::LYS2, ura3, leu2::hisG, his3::hisG, trp1::hisG, UME6-3V5-HIS3	SK1
FW2370	MATα, ho::LYS2, lys2, ura3, leu2::hisG, his3::hisG, trp1::hisG, ime1-t-NatMX4<br>MATa, ho::LYS2, lys2, ura3, leu2::hisG, his3::hisG, trp1::hisG, ime1-t-NatMX4	SK1
FW2449	Mata, ho::LYS2, lys2, ura3, leu2::hisG, his3::hisG, trp1::hisG, KanMX::pIME1-Δu6<br>MATα, ho::LYS2, lys2, ura3, leu2::hisG, his3::hisG, trp1::hisG, KanMX::pIME1-Δu6	SK1

FW2571	*MATa, ho::LYS2, lys2, ura3, leu2::hisG, his3::hisG, trp1::hisG, ime1-t-NatMX4, KanMX::pIME1-Δu6*<br>*MATα, ho::LYS2, lys2, ura3, leu2::hisG, his3::hisG, trp1::hisG, ime1-t-NatMX4, KanMX::pIME1-Δu6*	*SK1*
FW2270	*Mata, ho::LYS2, ura3, lys2, leu2::hisG, his3:hisG, trp1::hisG, ime1::pCUP-3HA-IME1-KanMX, TRP1-RME1-H*<br>*MATα, ho::LYS2, ura3, lys2, leu2::hisG, his3:hisG, trp1::hisG, ime1::pCUP-3HA-IME1-KanMX, TRP1-RME1-H*	*SK1*
FW3006	*Mata, ho::LYS2, ura3, lys2, leu2::hisG, his3:hisG, trp1::hisG, ime1::pCUP-3HA-IME1-KanMX*<br>*MATα, ho::LYS2, ura3, lys2, leu2::hisG, his3:hisG, trp1::hisG, ime1::pCUP-3HA-IME1-KanMX*	*SK1*
FW2842	*MATα, ho::LYS2, ura3, lys2, leu2::hisG, his3:hisG, trp1::hisG, ime1::pCUP-3HA-IME1-KanMX, HIS3::pIME1-Δu6*<br>*Mata, ho::LYS2, ura3, lys2, leu2::hisG, his3:hisG, trp1::hisG, ime1::pCUP-3HA-IME1-KanMX, HIS3::pIME1-Δu6*	*SK1*
FW2662	*MATa, ho::LYS2, lys2, ura3, leu2::hisG, his3::hisG, trp1::hisG, KanMX::pIME1-Δu6, TRP1-RME1-H*<br>*MATα, ho::LYS2, lys2, ura3, leu2::hisG, his3::hisG, trp1::hisG, KanMX::pIME1-Δu6, TRP1-RME1-H*	*SK1*
FW2449	*Mata, ho::LYS2, lys2, ura3, leu2::hisG, his3::hisG, trp1::hisG, KanMX::pIME1-Δu6*<br>*MATα, ho::LYS2, lys2, ura3, leu2::hisG, his3::hisG, trp1::hisG, KanMX::pIME1-Δu6*	*SK1*
FW5337	*MATa, ho::LYS2, lys2, ura3, leu2::hisG, his3::hisG, trp1::hisG, ime1Δ::HIS3, ura3::pIME1-LACZ-URA3*<br>*MATα, ho::LYS2, ura3, lys2, leu2::hisG, his3:hisG, trp1::hisG, ime1::pCUP-3HA-IME1-KanMX, TRP1-RME1-H*	*SK1*
FW5341	*MATa, ho::LYS2, lys2, ura3, leu2::hisG, his3::hisG, trp1::hisG, ime1Δ::HIS3, ura3::pIME1-Elt-LACZ-URA3*<br>*MATα, ho::LYS2, ura3, lys2, leu2::hisG, his3:hisG, trp1::hisG, pIME1::pCUP-3HA-IME1-KanMX, TRP1-RME1-H*	*SK1*

FW4653	*MATa, ho::LYS2, lys2, ura3, leu2::hisG, his3::hisG, trp1::hisG, pIME1-GFP-IME1*<br>*MATα, ho::LYS2, lys2, ura3, leu2::hisG, his3::hisG, trp1::hisG, pIME1-GFP-IME1*	*SK1*
FW5291	*MATa, ho::LYS2, lys2, ura3, leu2::hisG, his3::hisG, trp1::hisG, pime1Δ::KanMX-pCUP-IME1, TRP1-RME1-H*<br>*MATα, ho::LYS2, lys2, ura3, leu2::hisG, his3::hisG, trp1::hisG, pIME1-sfGFP-IME1*	*SK1*
FW5295	*MATa, ho::LYS2, lys2, ura3, leu2::hisG, his3::hisG, trp1::hisG, KanMX::pIME1-Δu6-GFP-IME1*<br>*MATα, ho::LYS2, lys2, ura3, leu2::hisG, his3::hisG, trp1::hisG, pime1Δ::KanMX-pCUP-IME1, TRP1-RME1-H*	*SK1*
FW4843	*MATa, ho::LYS2, lys2, ura3, leu2::hisG, his3::hisG, trp1::hisG, TRP1-RME1, pIME1-GFP-IME1, flo8Δ::KanMX*<br>*MATα, ho::LYS2, lys2, ura3, leu2::hisG, his3::hisG, trp1::hisG, NatMX::pIME1-Δirt2-mCherry-URA3, flo8Δ::KanMX,*	*SK1*
FW4844	*MATa, ho::LYS2, lys2, ura3, leu2::hisG, his3::hisG, trp1::hisG, TRP1-pRME1-Δaa1, pIME1-GFP-IME1, flo8Δ::KanMX*<br>*MATα, ho::LYS2, lys2, ura3, leu2::hisG, his3::hisG, trp1::hisG, NatMX::pIME1-Δirt2-mCherry-URA3, flo8Δ::KanMX,*	*SK1*
FW5051	*MATa, ho::LYS2, lys2, ura3, leu2::hisG, his3::hisG, trp1::hisG, pIME1-GFP-IME1, KanMX-pCUP-IRT2, TRP1-pRME1-Δaa1-RME1, flo8Δ::KanMX*<br>*MATα, ho::LYS2, lys2, ura3, leu2::hisG, his3::hisG, trp1::hisG, NatMX::pIME1-Δirt2-mCherry-URA3, flo8Δ::KanMX,*	*SK1*
FW1366	*MATa, ho::LYS2, lys2, ura3, leu2::hisG, his3::hisG, trp1::hisG, pime1Δ::KanMX-pCUP-IME1*<br>*MATα, ho::LYS2, lys2, ura3, leu2::hisG, his3::hisG, trp1::hisG, TRP1-RME1-H-3V5-HIS3*	*SK1*
FW1312	*MATa, ho::LYS2, ura3, leu2::hisG, his3::hisG, trp1::hisG, TRP1-RME1-H, set2Δ::HIS3, set3Δ::HIS3*<br>*MATα, ho::LYS2, ura3, leu2::hisG, his3::hisG, trp1::hisG, TRP1-RME1-H, set2Δ::HIS3, set3Δ::HIS3*	*SK1*

FW1208	MATa, ho::LYS2, ura3, leu2::hisG, his3::hisG, trp1::hisG, UME6-3V5-HIS3<br>MATα, ho::LYS2, ura3, leu2::hisG, his3::hisG, trp1::hisG, UME6-3V5-HIS3	SK1
FW5918	MATa, ho::LYS2, ura3, leu2::hisG, his3::hisG, trp1::hisG, UME6-3V5-HIS3, ime1Δ::HIS3<br>MAT□, ho::LYS2, ura3, leu2::hisG, his3::hisG, trp1::hisG, UME6-3V5-HIS3, ime1Δ::HIS3	SK1
FW5924	MATa, ho::LYS2, ura3, leu2::hisG, his3::hisG, trp1::hisG, TRP1-RME1-H, ime1-t-NatMX4, KanMX-pCUP-IRT2<br>MATα, ho::LYS2, ura3, leu2::hisG, his3::hisG, trp1::hisG, TRP1-RME1-H, ime1-t-NatMX4, KanMX-pCUP-IRT2	SK1
FW2241	MATa, ho::LYS2, ura3, leu2::hisG, his3::hisG, trp1::hisG, KanMX-pCUP-IRT2, TRP1-RME1-H-3V5-HIS3, set2Δ::HIS3, set3Δ::HIS3<br>MATα, ho::LYS2, ura3, leu2::hisG, his3::hisG, trp1::hisG, KanMX-pCUP-IRT2, TRP1-RME1-H-3V5-HIS3, set2Δ::HIS3, set3Δ::HIS3	SK1

Supplementary Table 2. Oligonucleotides sequence used

Oligo number	name	sequence	Used for:
FW463	IME1 FW	caacgcctccgataatgtatatg	Fig: Sup 7c
FW464	IME1 REV	acgtcgaaggcaatttctaatg	Fig: Sup 7c
FW481	IME1 -2000 FW	atttttagcgactgccgaaa	Fig: 1c, Sup 7a, Sup 7c
FW482	IME1 -2000 REV	atgcaacgcctacttgtttt	Fig: 1c, Sup 7a, Sup 7c
FW159	IME1 -1000 FW	gggtcttaaatacgcagggaat	Fig: Sup 7c
FW160	IME1 -1000 REV	ggcagttcaaaggcttttctta	Fig: Sup 7c
FW106	ACT1 FW	gtaccaccatgttcccaggtatt	Fig: 1c, Sup 7a, Sup 7c, Sup 5b
FW268	ACT1 3'end REV	agatggaccactttcgtcgt	Fig. Sup 5b
FW107	ACT1 REV	caagatagaaccaccaatccaga	Fig: 1c, Sup 7a, Sup 7c
FW329	IME1 -2100 FW	ccgtatggtgttggagtaatttg	Fig: 2b, 3b, 4b, Sup 4a
FW330	IME1 -2100 REV	tgccatttagtggacttcttgag	Fig: 2b, 3b, 4b, Sup 4a
FW43	HMR1 FW	acgatccccgtccaagttatg	Fig: 2b, 3b, 4b, Sup 4a
FW50	HMR1 REV	cttcaaaggagtcttaatttccctg	Fig: 2b, 3b, 4b, Sup 4a
FW490	IME1 -2400 FW	tgatatgtatgggttaaaaaggatg	Fig: 2a, 3d, 4c, 4d, Sup 2a, Sup 2b, Sup3e, Sup 4b, Sup 5c
FW540	IME1 -1000 REV	ggcagttcaaaggcttttctta	Fig: 2a, 3d, 4c, 4d, Sup 2a, Sup 2b, Sup 3e, Sup 4b, Sup 5c
FW489	IME1 -2400 REV	atgcaacgcctacttgtttt	Fig: 1b, 3c, Sup 1b, Sup 3b, Sup 3d
FW493	IME1 UME6Δ check FW	gatggagggttggcataaaa	Fig: 1b, 3c, Sup 1b, Sup 3b, Sup 3d
FW1555	IME1 TSS +201 FW	gctgcagaacttggttcataca	Fig: 2a, 3d, 4c, 4d, Sup 2a, Sup 2b, Sup 4b

FW464	IME1 REV	acgtcgaaggcaatttctaatg	Fig: 2a, 3d, 4c, 4d, Sup 2a, Sup 2b, Sup 4b
FW1895	nucleo 3 fw	aaaatgaaaggcagaagatg	Fig. 4a, Sup 5a
FW1896	nucleo 3 rev	ctggtatggtattgtaagga	Fig. 4a, Sup 5a
FW1897	nucleo 4 fw	cgtcacaaaaatcactcata	Fig. 4a, Sup 5a
FW1898	nucleo 4 rev	ggcggaaagaacagcatgat	Fig. 4a, Sup 5a
FW1290	nucleo 5 fw	aataccataccagccgcaag	Fig. 4a, Sup 5a
FW1291	nucleo 5 rev	ctccaacaccatacgggccgt	Fig. 4a, Sup 5a
FW1899	nucleo 6 fw	atcatgctgttctttccgcc	Fig. 4a, Sup 5a, Sup 5b
FW1900	nucleo 6 rev	cccacccttcttttattgag	Fig. 4a, Sup 5a, Sup 5b
FW1901	nucleo 7 fw	attaatgtattccctcacgg	Fig. 4a, Sup 5a
FW1902	nucleo 7 rev	cttcttgaggttcttttgacatc	Fig. 4a, Sup 5a
FW1903	nucleo 8 fw	aagaagggtgggggtgtatt	Fig. 4a, Sup 5a
FW1904	nucleo 8 rev	tcgctaaaaatgtaagcgcg	Fig. 4a, Sup 5a
FW1087	IME1 -2050 fw	ggatgtcaaaagaacctcaaga	Fig. 4a, Sup 5a
FW1088	IME1 -2050 rev	tttcggcagtcgctaaaaat	Fig. 4a, Sup 5a
FW1905	nucleo 9 fw	tcaagaagtccactaaatgg	Fig. 4a, Sup 5a
FW1906	nucleo 9 rev	acaattttatgcttttgagg	Fig. 4a, Sup 5a
FW1907	nucleo 10 fw	gccgaaaacgtacggctaac	Fig. 4a, Sup 5a
FW1908	nucleo 10 rev	gctcactttttttcctaccca	Fig. 4a, Sup 5a
FW1909	nucleo 11 fw	aaaacaagtaggcgttgcat	Fig. 4a, Sup 5a, Sup 5b
FW1910	nucleo 11 rev	accctatttcttcacgaggg	Fig. 4a, Sup 5a, Sup 5b
FW1030	nucleo 12 fw	gagcgccaacactatataag	Fig. 4a, Sup 5a
FW1031	nucleo 12 rev	caaattctttaaactaagcgc	Fig. 4a, Sup 5a
FW1911	nucleo 13 fw	gggtcctgcattgatatttt	Fig. 4a, Sup 5a
FW1912	nucleo 13 rev	gtacacattctttctccaag	Fig. 4a, Sup 5a

FW1913	nucleo 14 fw	gcgcttagtttaaagaatttg	Fig. 4a, Sup 5a
FW1914	nucleo 14 rev	cctttgttttctctttatcccc	Fig. 4a, Sup 5a
FW1915	nucleo 15 fw	cttgattattggcattccgc	Fig. 4a, Sup 5a
FW1916	nucleo 15 rev	ttgctcggaggtactagtca	Fig. 4a, Sup 5a
FW1917	nucleo 16 fw	ggggataaagagaaaacaaagg	Fig. 4a, Sup 5a
FW1918	nucleo 16 rev	tctctttttacatctgttcgc	Fig. 4a, Sup 5a
FW1841	Scr1 fw	gaagtgtcccggctataataaa	Fig. 1b, 3c, 4d, Sup 1b, Sup 3e, Sup 5c
FW1842	Scr1 Rev	gacgctggataaaactcccc	Fig. 1b, 3c, 4d, Sup 1b, Sup 3e, Sup 5c

Supplementary References

1. Lardenois, A. *et al.* Execution of the meiotic noncoding RNA expression program and the onset of gametogenesis in yeast require the conserved exosome subunit Rrp6. *Proc Natl Acad Sci U S A* **108**, 1058-63 (2011).
2. Robinson, J.T. *et al.* Integrative genomics viewer. *Nat Biotechnol* **29**, 24-6 (2011).
3. Wery, M. *et al.* Nonsense-Mediated Decay Restricts LncRNA Levels in Yeast Unless Blocked by Double-Stranded RNA Structure. *Mol Cell* **61**, 379-392 (2016).
